# Utilisation of Genetics Services for Residents at ‘Dar Tal-Providenza’, Malta Under the Care of the Intellectual Disability Clinic

**DOI:** 10.1192/bjo.2026.11665

**Published:** 2026-06-30

**Authors:** Nicole Mifsud, Noemi Zammit Cortis, Jean-Pierre Giorgio

**Affiliations:** 1 Mater Dei Hospital, Msida, Malta; 2 Mental Health Services, Attard, Malta

## Abstract

**Aims:**

This audit aimed to determine the proportion of adults with Intellectual Disability (ID) residing at ‘Dar tal-Providenza’ residence, who had undergone genetic testing confirming a genetic diagnosis, assess documentation of genetic testing discussions or referrals, and evaluate alignment of current practice with national and international guidelines for genetic testing in neurodevelopmental disorders.

**Methods:**

All adults under the care of the ID team with a documented diagnosis of ID and/or Autism Spectrum Disorder (ASD) were included. Electronic Patient Records were reviewed for demographic data, primary diagnoses, documentation of genetic testing or referral to genetics services, discussions with patients or carers, and documentation of results and follow-up actions in accordance with National Institute for Health and Care Excellence (NICE) and European Society of Human Genetics (ESHG) guidelines.

**Results:**

The cohort consisted of 80 adults aged ≥18 years, with a mean age of 50.4 years and a gender distribution of 49 males and 31 females. Most individuals had multiple co-existing neurodevelopmental, neurological, genetic, and mental health diagnoses. ID severity was mild–moderate in 23.75% (n=19), moderate–severe in 57.5% (n=46), and unspecified in 18.75% (n=15). An associated genetic syndrome was identified in 16.25% (n=13).

ASD was the second most common diagnosis (22.5%, n=18) and a frequent comorbidity with ID (16.25%, n=13). Other diagnoses included cerebral palsy (13.75%, n=11), trisomy 21 (11.25%, n=9), epilepsy (6.25%, n=5), Fragile X syndrome, Cri-du-Chat syndrome, and Peters-plus syndrome (each 1.25%, n=1). Challenging behaviour co-occurred in 8.75% (n=7). Mental health comorbidity was common, including bipolar affective disorder (6.25%, n=5), emotionally unstable personality disorder, post-traumatic stress disorder, schizoaffective disorder, anxiety disorder, and psychotic depression (each 1.25%, n=1).

Only 6 patients (7.5%) had a documented genetics referral, with genetic testing discussed in one case (1.25%). Among those referred, five had confirmed diagnoses, one was awaiting results, and no follow-up plans were documented.

**Conclusion:**

This audit identified a highly complex cohort with extensive neurodevelopmental and mental health needs. Despite meeting NICE and ESHG criteria, genetics referral was low (7.5%) with minimal documented discussion or follow-up, highlighting the need for further training and collaboration between the Psychiatry and Genetics Department in Malta.

